# Case Report: Giant L1 dumbbell-shaped spinal schwannoma with osteolytic destruction and intense [^18^F]FDG uptake on PET/CT mimicking a malignant tumor

**DOI:** 10.3389/fmed.2026.1801641

**Published:** 2026-04-21

**Authors:** Chenghao Jiang, Dawei Wang, Jianqiang Xing, Bin Shao, Yongfeng Dou

**Affiliations:** Department of Spinal Surgery, Binzhou Medical University Hospital, Binzhou, China

**Keywords:** [18F]FDG PET/CT, case report, dumbbell-shaped tumor, instrumented fusion, lumbar spine, osteolytic destruction, schwannoma

## Abstract

**Background:**

Giant dumbbell-shaped intraspinal–extraspinal schwannomas with osteolytic destruction and intense [^18^F]FDG uptake may mimic malignant tumors, creating diagnostic uncertainty and substantial surgical challenges related to neural decompression, maximal safe resection, and spinal stabilization.

**Case presentation:**

A 63-year-old woman presented with a 5-month history of severe low back pain and left-sided radiculopathy. Neurological examination revealed decreased pinprick sensation over the left lower leg and foot, mild weakness of left great toe dorsiflexion and plantarflexion [Medical Research Council (MRC) grade 4/5], a diminished left ankle reflex, and a positive left straight-leg raise at 40°, without sphincter dysfunction. Dynamic contrast-enhanced lumbar MRI demonstrated a lobulated, heterogeneously enhancing mass with cystic/necrotic components centered at the left L1 posterior element-pedicle region, with adjacent osteolytic destruction and spinal canal compromise. Preoperative [^18^F]FDG PET/CT showed an irregular dumbbell-shaped lesion extending across the paravertebral, foraminal, and intraspinal compartments with heterogeneous [^18^F]FDG uptake (SUVmax 10.2), without additional suspicious skeletal or visceral lesions. The patient underwent a posterior-only, single-stage resection of both the epidural intraspinal and large extraspinal components, followed by long-segment instrumented fusion (T11–L3) and posterolateral grafting because of tumor-related pedicle/vertebral destruction and iatrogenic instability. Histopathology confirmed schwannoma with S-100 and SOX10 positivity and a low Ki-67 index (~3%). Postoperatively, pain and radicular symptoms improved with neurological recovery; radiographs demonstrated satisfactory implant position without evidence of screw loosening.

**Conclusion:**

Spinal schwannomas can exhibit intense [^18^F]FDG uptake and osteolytic changes yet remain benign. For selected destructive dumbbell-shaped schwannomas spanning the paravertebral, foraminal, and intraspinal compartments, a posterior-only, single-stage approach may achieve effective decompression and facilitate immediate stabilization in selected patients when anatomical accessibility and mechanical considerations are favorable.

## Introduction

Schwannomas are common benign peripheral nerve sheath tumors and frequently arise in the spinal canal, typically presenting as well-circumscribed lesions amenable to surgical resection. However, schwannomas may occasionally reach a large size and exhibit intense and/or heterogeneous [^18^F]FDG uptake on PET/CT, imaging features that are more often associated with malignant peripheral nerve sheath tumors (MPNSTs) or other aggressive neoplasms, thereby creating diagnostic uncertainty (1). In particular, some schwannomas demonstrate extensive intraspinal–extraspinal dumbbell-shaped extension through the neural foramen, accompanied by cystic degeneration, hemorrhage, and progressive bone remodeling or destruction, which can further obscure preoperative risk stratification and broaden the differential diagnosis (2–4).

When imaging reveals osteolytic changes together with intense or heterogeneous [^18^F]FDG avidity, benign schwannoma may be mistaken for low-grade malignancy or a musculoskeletal-origin tumor, complicating preoperative counseling and management planning (2–4). Importantly, diagnostic pitfalls are not limited to [^18^F]FDG. Schwannomas have also been reported as incidental or misleading foci on choline-based PET/CT, including cases highlighted as diagnostic pitfalls on both [^18^F]choline PET/CT and [^18^F]FDG PET/CT, underscoring that tracer uptake alone is insufficient to infer malignancy (5, 6). Consistent with this concept, clinical reports indicate that PET findings may incorrectly suggest metastatic disease or malignant soft-tissue tumors in the presence of benign schwannomas (7, 8).

Moreover, giant dumbbell lesions spanning the paravertebral, foraminal, and intraspinal compartments require meticulous surgical planning to balance maximal safe resection, neural decompression, and durable spinal stability. Here, we report a giant L1 dumbbell-shaped schwannoma with osteolytic destruction and high [^18^F]FDG uptake (SUVmax 10.2) treated using a posterior-only, single-stage approach with long-segment instrumented fusion, and we discuss key diagnostic pitfalls and surgical considerations relevant to contemporary spine oncology practice.

## Case description

### Patient information

A 63-year-old woman with a 7-year history of hypertension, well controlled with antihypertensive medication, presented with a 5-month history of severe low back pain with left-sided radicular pain radiating from the left buttock to the posterior thigh and foot. The pain was persistent, aggravated by coughing and exertion, and partially relieved by rest. She denied fever, night sweats, unintentional weight loss, gait instability, or bowel/bladder dysfunction. Her medical history was otherwise unremarkable, with no prior surgery, malignancy, known tuberculosis exposure, or relevant family history.

### Clinical findings

On examination, lumbar range of motion was limited. There was midline tenderness over the lower lumbar spinous processes and deep tenderness of the left paraspinal muscles, reproducing radicular pain. Neurological examination demonstrated decreased pinprick sensation over the left lower leg and foot; mild weakness of left great toe dorsiflexion and plantarflexion [Medical Research Council (MRC) grade 4/5] with otherwise normal strength (5/5) in both lower extremities; a diminished left ankle reflex with preserved bilateral knee reflexes; and a positive left straight-leg raise at 40° with a positive crossed/augmentation (reinforcement) maneuver. No pathological reflexes were elicited. Perianal sensation, anal sphincter tone, and anal reflex were intact.

### Diagnostic assessment

#### Dynamic contrast-enhanced MRI

**Figure 1:**
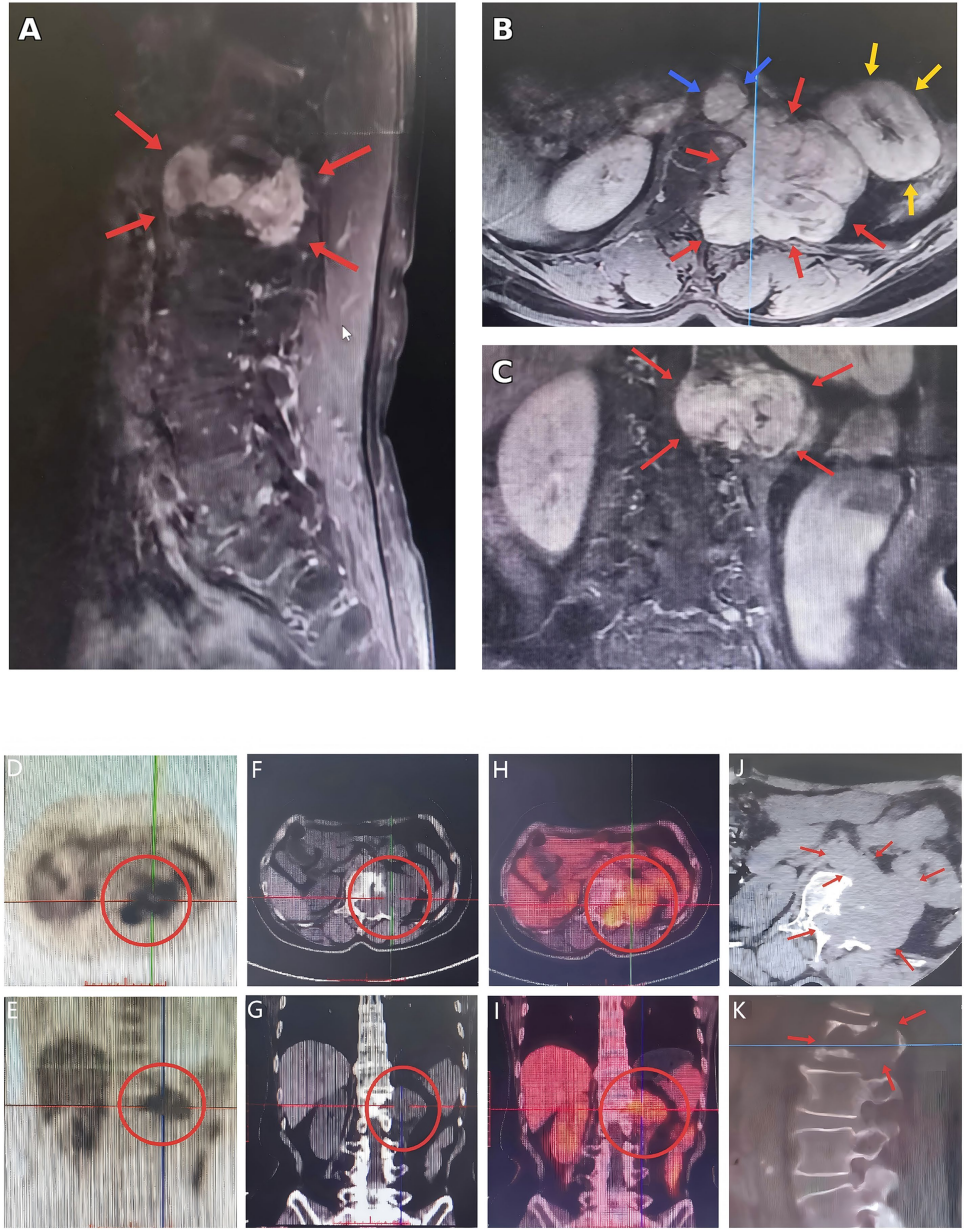
Preoperative contrast-enhanced MRI and [^18^F]FDG PET/CT with CT characterization of a giant dumbbell-shaped schwannoma at the left L1 level. **(A)** Sagittal contrast-enhanced MRI demonstrates a large, heterogeneously enhancing mass centered at the left L1 pedicle/posterior element region with intraspinal extension and spinal canal compromise. **(B)** Axial MRI depicts the paravertebral–foraminal–intraspinal components of the lesion and its relationship to adjacent retroperitoneal structures. **(C)** Coronal MRI further delineates the extraforaminal/paravertebral extent of the tumor. Red arrows indicate the schwannoma; blue arrows indicate the abdominal aorta; yellow arrows indicate the left kidney. **(D,E)** Maximum-intensity projection (MIP) and multiplanar PET views demonstrate focal [^18^F]FDG uptake corresponding to the lesion (red circles). **(F,G)** Non-contrast CT reconstructions show a destructive mass centered at the left L1 posterior elements/vertebral body with osteolytic erosion and extension toward the spinal canal and paravertebral space (red circles). **(H,I)** Fused PET/CT images confirm spatial concordance between increased [^18^F]FDG activity and the CT-defined mass (red circles). **(J,K)** Additional diagnostic CT images highlight osseous involvement and mass effect at the left L1 level (red arrows). Overall, the imaging findings are consistent with a dumbbell-shaped lesion spanning the paravertebral, foraminal, and intraspinal compartments.

Dynamic contrast-enhanced lumbar MRI demonstrated a lobulated soft-tissue mass at the left posterior element/pedicle region of L1 with low-to-intermediate signal intensity on T1-weighted images and high signal intensity on T2-weighted images, containing internal cystic/necrotic components. Post-contrast images showed marked heterogeneous enhancement. Adjacent osseous destruction was present, and the lesion extended toward the spinal canal, resulting in canal compromise and compression of the thecal sac/neural elements at the thoracolumbar junction. Concomitant degenerative findings included mild anterolisthesis above L4, Schmorl’s nodes at L2–L3, and disc bulging/herniation at L4/5 and L5/S1 (Figures 1A–C).

#### Non-contrast CT (thoracic/thoracolumbar region)

Non-contrast CT of the thoracic/thoracolumbar spine revealed a large soft-tissue mass centered at the left L1 posterior element/pedicle region, bulging leftward toward the abdominal cavity and exerting mass effect on the left kidney. The lesion abutted the aorta and showed heterogeneous internal attenuation. Osteolytic destruction involved the left aspect of the L1 vertebral body and adjacent posterior elements, with a maximal dimension of approximately 6.4 × 8.1 cm. The mass partially protruded into the spinal canal, producing a space-occupying effect and compression of the thecal sac/neural elements (Figures 1J,K).

Thoracic alignment was preserved with a maintained physiologic curvature. Degenerative changes included marginal osteophytes and focal endplate depressions compatible with Schmorl’s nodes. No definite thoracic disc bulge or herniation was identified, and the surrounding paraspinal soft tissues were unremarkable.

#### [^18^F]FDG PET/CT

[^18^F]FDG PET/CT demonstrated an irregular dumbbell-shaped soft-tissue mass at the left aspect of L1 extending across the paravertebral region, left neural foramen, and intraspinal canal, with osteolytic destruction and peripheral sclerosis. The lesion showed heterogeneous [^18^F]FDG uptake (SUVmax ~10.2) with low-attenuation necrotic components. Foraminal widening at T12/L1 and canal narrowing at L1 were noted. No additional abnormal [^18^F]FDG-avid osseous or visceral lesions were identified. Incidental findings included thyroid nodules with mild [^18^F]FDG uptake and a small right middle-lobe pulmonary nodule without hypermetabolism; outpatient follow-up was recommended (Figures 1D–I).

### Preoperative impression and differential diagnosis

Given the osteolytic destruction and intense [^18^F]FDG avidity, the leading consideration was a peripheral nerve sheath tumor (PNST), with schwannoma favored; however, a low-grade malignant PNST could not be excluded. The differential diagnosis also included a musculoskeletal-origin tumor and metastatic disease, while infection was considered less likely in the absence of systemic symptoms of inflammation. In view of symptomatic spinal canal compromise and progressive functional limitation, surgical decompression and resection were indicated, with definitive diagnosis to be established by postoperative histopathology.

### Therapeutic intervention (surgery)

On February 17, 2023, the patient underwent a posterior midline approach under general anesthesia in the prone position. A midline incision from T11 to L3 was performed with subperiosteal exposure of the posterior elements. Under fluoroscopic guidance, pedicle screws were inserted bilaterally at T11/T12 and L2/L3, with an additional screw placed at L1 on the right side; the left L1 pedicle was compromised by tumor-related destruction and was therefore not instrumented. A left-sided L1 laminectomy with partial resection of the involved posterior elements was performed. An epidural intraspinal mass was identified medial to the left L1 pedicle, measuring approximately 2.5 × 3.0 × 2.5 cm. The lesion appeared yellow and lobulated with cystic/necrotic areas and extended through the neural foramen. The large extraspinal component (approximately 9 × 8 × 10 cm) was resected piecemeal. After macroscopic tumor removal, the operative field was carefully inspected, and no gross residual tumor was identified.

Precontoured rods were then applied and secured to achieve stable fixation and maintain decompression; the thecal sac and neural elements were free of compression. Posterolateral bone grafting was performed using 21 cm^2^ of allograft. Estimated blood loss was 500 mL. The patient received 3.5 units of leukocyte-reduced packed red blood cells intraoperatively. A closed-suction drain was placed, and the wound was closed in layers.

### Pathological findings

**Figure 2:**
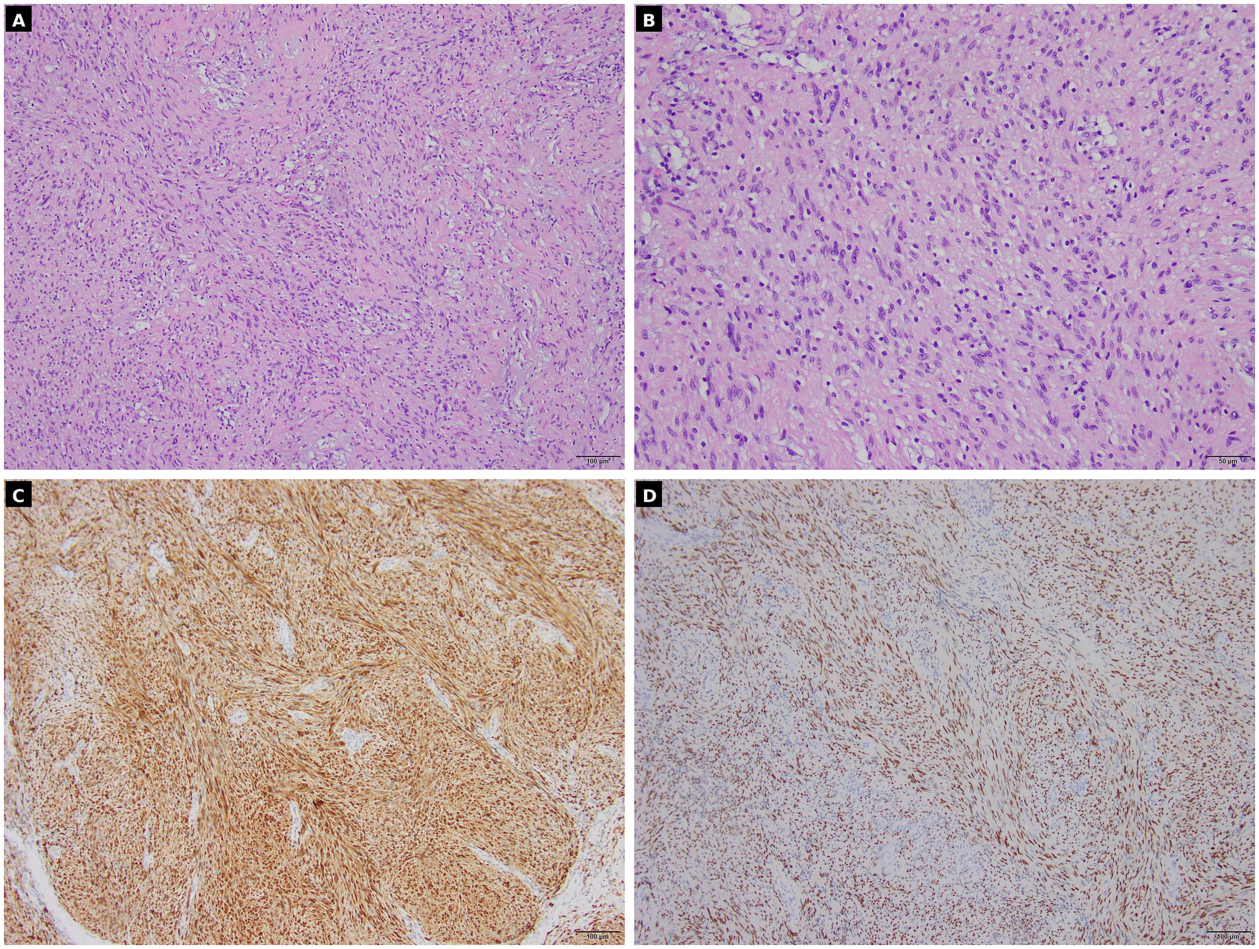
Histopathology of schwannoma. **(A,B)** H&E staining (100 × and 200×). **(C,D)** Immunohistochemistry demonstrating diffuse positivity for S-100 and SOX10. The Ki-67 proliferation index was approximately 3%.

Gross examination revealed fragmented gray-white to yellowish tissue fragments measuring 11 × 10 × 3 cm in aggregate, with a firm cut surface. Histopathological evaluation was consistent with schwannoma. Immunohistochemistry demonstrated vimentin positivity, diffuse strong S-100 positivity, and SOX10 positivity; CD34, epithelial membrane antigen (EMA), smooth muscle actin (SMA), and neurofilament (NF) were negative, while desmin showed focal positivity. The Ki-67 proliferation index was approximately 3%, supporting a benign peripheral nerve sheath tumor (Figure 2). Overall, the histomorphology and immunophenotype were in keeping with a benign schwannoma.

### Follow-up and outcomes

At postoperative day (POD) 10, the patient reported marked clinical improvement. Interspinous tenderness and deep tenderness of the left paraspinal muscles were substantially reduced compared with the preoperative status, and left-sided radicular pain was significantly relieved, allowing resumption of normal daily activities. Neurological examination demonstrated objective recovery: pinprick sensation over the left lower leg and foot improved with less hypoesthesia; motor strength of left great toe dorsiflexion and plantarflexion increased to MRC 5/5 (from 4/5 preoperatively), with otherwise full strength in both lower extremities. The left ankle reflex recovered to normal, while bilateral knee reflexes remained normal. The left straight-leg raise improved from 40° (positive) to 60° (mildly positive), and the reinforcement maneuver became negative; the right straight-leg raise remained negative at 70°. No pathological reflexes were elicited. Perianal sensation, anal sphincter tone, and anal reflex were preserved, with no bowel or bladder dysfunction.

**Figure 3:**
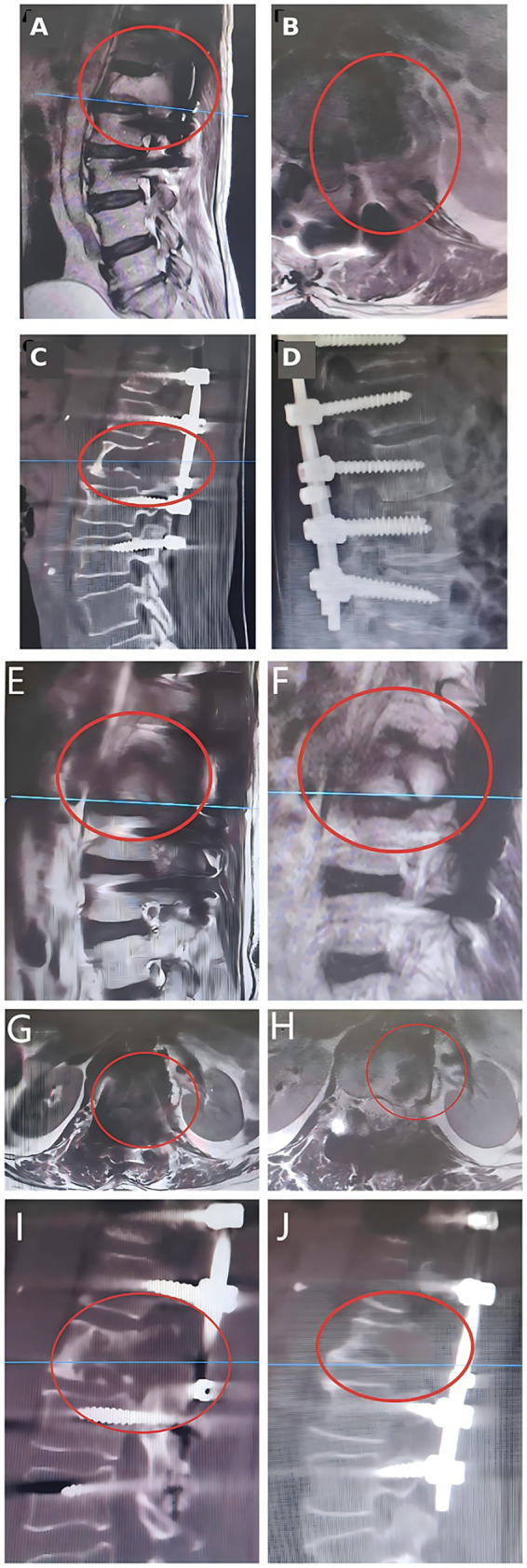
Early postoperative imaging (postoperative day 10) and follow-up imaging at 3 months and 1 year. **(A)** Sagittal lumbar MRI demonstrates expected postoperative changes after resection of the L1 lesion with T11–L3 posterior instrumentation; postoperative fluid/edema is present within the surgical bed (circle). **(B)** Corresponding axial MRI at the L1 level shows a mass-like postoperative signal abnormality within the left paravertebral/foraminal operative region (circle), compatible with early postoperative cavity/fluid and reactive change; residual lesion cannot be excluded on the non-contrast protocol. **(C)** Sagittal CT confirms postoperative osseous changes at L1 and the position of the posterior fixation construct. **(D)** Lateral radiograph shows satisfactory alignment and implant position without evidence of hardware failure. **(E,F)** Sagittal MRI at 3 months **(E)** and 1 year **(F)** demonstrates interval evolution of the surgical bed at the operated level (circle). **(G,H)** Corresponding axial MRI at 3 months **(G)** and 1 year **(H)** depicts the local postoperative anatomy at the same level (circle). **(I,J)** CT at 3 months **(I)** and 1 year **(J)** provides complementary assessment of osseous remodeling and postoperative stability (circle).

At POD 10, thoracolumbar MRI/CT demonstrated expected postoperative changes, including T11–L3 instrumentation, surrounding soft-tissue edema, and postoperative gas. On early non-contrast MRI, a mass-like T2-hyperintense signal at the left L1 region (maximal axial dimension approximately 7.0 × 3.7 cm) was reported and was interpreted as most consistent with postoperative cavity/fluid and reactive change, although residual lesion could not be definitively excluded; interval follow-up imaging was therefore warranted. Plain radiographs demonstrated satisfactory implant position without evidence of screw loosening (Figures 3A–D).

At 3 months, MRI/CT showed a stable postoperative appearance without new intraspinal compression. At 1-year follow-up, MRI demonstrated interval decrease in the perilesional abnormal signal around the left L1 region compared with the 3-month study, with no progressive mass effect or new canal compromise, supporting no radiologic progression during follow-up (Figures 3E–J). Continued long-term clinical and radiologic surveillance was recommended to monitor for delayed local recurrence and late instrumentation-related complications beyond the 1-year time point.

A summary of the diagnostic work-up and treatment timeline is provided in Table 1.

**Table 1:** Timeline of clinical course, diagnostic evaluation, treatment, and follow-up.

Date/Timepoint	Event
~Sep 2022	Onset of low back pain with left-sided radicular symptoms.
Jan 30, 2023	Dynamic contrast-enhanced lumbar MRI: Destructive mass centered at the left L1 pedicle/posterior element region with cystic/necrotic components and heterogeneous enhancement; focal intraspinal extension causing spinal canal compromise and compression of the thecal sac/neural elements. Concomitant degenerative lumbar findings were present.
Jan 31, 2023	Hospital admission for suspected spinal tumor.
Preoperative (early Feb 2023)	[^18^F]FDG PET/CT: Irregular dumbbell-shaped lesion spanning the paravertebral, foraminal, and intraspinal compartments; heterogeneous [^18^F]FDG uptake (SUVmax 10.2). No additional abnormal [^18^F]FDG-avid skeletal or visceral lesions were identified.
Feb 17, 2023	Posterior-only, single-stage tumor resection with T11–L3 instrumented fusion and posterolateral allograft grafting.
Postoperative day 10 (Feb 26, 2023)	MRI (non-contrast, thoracolumbar): Expected postoperative changes with surrounding edema; mass-like T2-hyperintense signal at the L1 surgical bed/left operative region (~7.0 × 3.7 cm on axial images), interpreted as early postoperative cavity/fluid and reactive change, although residual lesion could not be excluded on the non-contrast protocol; interval follow-up was warranted. CT: Postoperative changes with residual bony defect at L1 and adjacent soft-tissue swelling/air. X-ray: Satisfactory implant position without screw loosening. Clinical status: Pain and radicular symptoms improved, with stable/improving neurological examination.
3-month follow-up	MRI (non-contrast): Stable postoperative appearance at L1 with ill-defined edema-like signal around the surgical bed; no progressive mass effect or new intraspinal compression. CT: Expected postoperative bony defect/remodeling at L1 with stable instrumentation-related findings.
1-year follow-up	MRI (non-contrast): Decreased abnormal signal around the L1 surgical bed compared with the 3-month study; no progressive mass effect or new intraspinal compression, supporting no radiologic progression during follow-up. CT: Stable postoperative osseous findings at L1 without new aggressive progression compared with 3-month CT.

## Discussion

### Diagnostic pitfall: [^18^F]FDG-avid schwannoma with destructive features can mimic malignancy

Giant spinal schwannomas may present with aggressive-appearing imaging features, including osteolytic destruction, heterogeneous enhancement, and marked [^18^F]FDG avidity, which can closely resemble low-grade malignancy or malignant peripheral nerve sheath tumor (MPNST). Importantly, [^18^F]FDG uptake intensity alone is not a reliable discriminator between benign and malignant peripheral nerve sheath lesions. In a cohort of pathology-proven schwannomas, [^18^F]FDG uptake demonstrated substantial inter-lesional variability, and “worrisome” PET/MRI features—such as heterogeneous uptake, internal non-enhancement/necrotic components, irregular margins, and perilesional edema—were observed in benign lesions and may mimic malignant behavior (1). Similarly, benign solitary schwannomas have been reported across a broad SUVmax spectrum (e.g., SUVmax 1.5–11.0), reinforcing that high SUV values can fall within the benign range and may lead to false-positive interpretations when considered in isolation (9). Consistent case-based reports across anatomic sites further support this concept, describing benign schwannomas that mimic metastatic disease or malignant soft-tissue tumors and thereby increase diagnostic uncertainty (10–12).

Functional imaging pitfalls are not limited to [^18^F]FDG. Benign schwannomas may demonstrate uptake on multiple tracers, including choline-based PET, which can introduce additional diagnostic confusion in oncologic workflows (5). Similar tracer-uptake variability has also been reported in intracranial/cavernous locations (6). Beyond the spine, benign schwannomas in other organs (e.g., adrenal) have been reported with [^18^F]FDG uptake sufficient to raise concern for malignancy (13).

In the present case, the combined preoperative phenotype—osteolytic destruction on CT, heterogeneous enhancement with cystic/necrotic components on MRI, and intense [^18^F]FDG uptake on [^18^F]FDG PET/CT (SUVmax 10.2)—reasonably prioritized MPNST and other invasive neoplasms in the differential diagnosis and supported an upfront surgical strategy with careful postoperative imaging surveillance. Nevertheless, postoperative histopathology confirmed schwannoma with S-100 and SOX10 positivity and a low Ki-67 index (~3%), underscoring that even markedly [^18^F]FDG-avid, destructive-appearing schwannomas may remain biologically benign and that definitive classification must rely on histopathology (Figure 2).

The biologic basis of high [^18^F]FDG uptake in benign schwannoma is likely multifactorial. Increased uptake may reflect regional hypercellularity, particularly Antoni A–predominant areas, variable metabolic demand within mixed solid and degenerative components, and microenvironmental factors such as inflammation, reparative change, or hemorrhagic/cystic degeneration. Therefore, metabolically intense or heterogeneous [^18^F]FDG uptake should be interpreted cautiously, especially when the proliferative index is low and histopathologic features remain benign, as in the present case.

### PET/CT: valuable for extent mapping and staging, but limited for definitive grading

Although [^18^F]FDG PET/CT provides clinically meaningful metabolic information, its most reproducible role in complex spinal tumors is often delineation of disease extent and systemic staging, rather than definitive benign-versus-malignant classification. In high-risk settings (e.g., neurofibromatosis type 1 with suspected malignant transformation), diagnostic accuracy studies support the use of SUV-based thresholds and composite algorithms for risk stratification; however, these approaches inevitably involve trade-offs between sensitivity and specificity, and false-positive [^18^F]FDG avidity remains a recognized limitation (3). A meta-analytic/Bayesian perspective further emphasizes that PET interpretation should integrate pretest probability and clinical context, and that SUV cutoffs cannot eliminate overlap between benign peripheral nerve sheath tumors (including schwannomas) and MPNST; therefore, histopathology remains the reference standard for final grading (14).

Complementary MRI-based approaches—including advanced quantitative methods such as radiomics and machine-learning models—may improve preoperative risk stratification, but they remain adjunctive and do not replace pathology when imaging is aggressive-appearing or metabolically avid (4). However, these methods were not applied in the present retrospective single case and therefore should be regarded as future directions rather than validated evidence from this report. In our patient, [^18^F]FDG PET/CT clarified the dumbbell-shaped continuity across the paravertebral, foraminal, and intraspinal compartments and, importantly, did not identify additional abnormal [^18^F]FDG-avid skeletal or visceral lesions, supporting a localized process. Thus, PET/CT contributed primarily to operative planning and exclusion of systemic disease, while the definitive diagnosis relied on postoperative histopathology.

### Postoperative imaging pitfall: early “mass-like” T2 hyperintensity can mimic residual disease

A key interpretive challenge in this case was the potential false-positive appearance on ultra-early postoperative MRI. At postoperative day 10, non-contrast MRI demonstrated a mass-like T2-hyperintense signal within the operative field, which may resemble the preoperative lesion and be misinterpreted as residual tumor or early recurrence. However, when postoperative timing and signal characteristics are considered, such findings are often more consistent with reactive postoperative changes, including tissue edema, inflammatory exudate, granulation tissue, and evolving hematoma/seroma, which can produce T2 hyperintensity and a pseudo–mass-like morphology in the early postoperative period. Overreliance on a single early MRI to label “residual disease” may therefore contribute to overtreatment and unnecessary patient anxiety. In lesions with aggressive-appearing preoperative imaging, early postoperative MRI is best used for baseline documentation and assessment of complications, whereas evaluation for residual or recurrent disease should be based on serial interval imaging rather than a single early time point.

In the present case, longitudinal follow-up provided a time-resolved evidentiary chain supporting this interpretation. At 3 months, postoperative imaging remained stable without interval enlargement or new compressive findings. At 1 year, the previously noted abnormal signal had substantially decreased, indicating that the early postoperative mass-like signal was more likely attributable to reversible postoperative reaction than persistent tumor tissue. Thus, a structured time-series interpretation—short-term stability with long-term regression—offers a pragmatic criterion favoring postoperative change over true residual/recurrent disease and may reduce misclassification and unnecessary interventions.

### Surgical strategy and stabilization: posterior-only one-stage resection with long-segment fixation in selected destructive dumbbell tumors

Surgical management of giant dumbbell-shaped schwannomas requires balancing maximal safe resection, neural decompression, and immediate mechanical stability. Approach selection should be individualized based on the relative intraspinal versus extraspinal tumor burden, accessibility of the paravertebral component through a posterior corridor, and the anticipated instability resulting from tumor-related bone destruction and required bony resection. Case-based surgical discussions have suggested that extensive extraspinal components and limited lateral exposure may favor combined or staged strategies, whereas selected tumors with feasible foraminal access may be amenable to a posterior-only approach (15, 16).

In our patient, a posterior-only, single-stage strategy enabled decompression of the epidural intraspinal component and piecemeal removal of the large extraspinal extension through the foraminal corridor within a single operative field. Given tumor-related pedicle/vertebral destruction and anticipated iatrogenic instability following posterior element resection, long-segment instrumented fusion (T11–L3) was selected to restore stability and protect the thecal sac/neural elements. When malignant transformation remains a meaningful preoperative concern, the distinct prognostic and treatment implications of MPNST further justify careful counseling, comprehensive imaging assessment, and a stabilization plan that anticipates both osseous compromise and surgical destabilization (2, 17). Postoperative clinical improvement, stable instrumentation, and the regressive trend on long-term imaging support posterior-only resection with immediate stabilization as a practical option for selected destructive spinal dumbbell schwannomas when anatomical accessibility permits.

Preoperative biopsy also warrants consideration in aggressive-appearing spinal peripheral nerve sheath tumors. In selected cases, image-guided biopsy may refine preoperative risk stratification and help distinguish among peripheral nerve sheath tumor, metastasis, primary bone/soft-tissue neoplasm, and infection. However, in the present patient, progressive radicular symptoms, neural compression, substantial osseous destruction, and anticipated mechanical instability already supported operative decompression and stabilization irrespective of final pathology. Accordingly, preoperative biopsy was unlikely to alter the need for surgery, although it may be valuable in other scenarios in which oncologic sequencing, neoadjuvant treatment, or the extent of resection would depend more directly on tissue diagnosis.

### Limitations and implications for practice

This report has several limitations inherent to a single-case design. First, although multimodal imaging was performed, advanced quantitative approaches such as radiomics, PET texture analysis, or comparison with standardized SUV thresholds in control cohorts were not available for this retrospective case; therefore, our observations remain primarily descriptive rather than algorithmically validated. Second, postoperative recovery was assessed mainly by neurological examination, symptom relief, and serial imaging, because standardized patient-reported outcome measures such as pain scores, disability indices, or quality-of-life instruments were not systematically recorded. Third, although 1-year follow-up demonstrated no radiologic progression and stable instrumentation, longer surveillance remains important to monitor for delayed recurrence and late mechanical complications. Fourth, the successful posterior-only single-stage strategy in this patient should not be interpreted as evidence of superiority over combined or staged approaches, as surgical planning for giant destructive dumbbell tumors must remain individualized. Finally, because definitive diagnosis was established after resection, this case cannot determine the optimal preoperative diagnostic pathway for similar lesions, including the selective role of image-guided biopsy.

## Conclusion

This case underscores that giant dumbbell-shaped spinal schwannomas may mimic malignant or musculoskeletal tumors when accompanied by osteolytic destruction and intense, heterogeneous [^18^F]FDG uptake on [^18^F]FDG PET/CT. In this setting, tracer avidity and bone erosion alone are insufficient to infer malignancy; instead, integrated interpretation of MRI/CT morphology, metabolic imaging for extent mapping and staging, and clinicoradiologic context is essential. For selected extensive paravertebral–foraminal–intraspinal lesions with structural compromise, a posterior-only, single-stage approach may achieve effective decompression and facilitate immediate stabilization when anatomical accessibility permits. Because this is a single-case report without advanced quantitative imaging analysis, standardized patient-reported outcome measures, or extended long-term follow-up, broader conclusions regarding optimal diagnostic algorithms or surgical superiority should be made cautiously.

## Data Availability

The original contributions presented in the study are included in the article/supplementary material, further inquiries can be directed to the corresponding authors.
